# Effect of capecitabine as monotherapy for HER2 normal metastatic breast cancer

**DOI:** 10.1007/s12032-024-02356-y

**Published:** 2024-03-27

**Authors:** Anne-Dorthe Mosgaard Knudsen, Mikala Wej Modvig, Marianne Vogsen, Annette Raskov Kodahl

**Affiliations:** 1 Department of Oncology, Odense University Hospital, Odense, Denmark; 2 Department of Clinical Research, University of Southern Denmark, Odense, Denmark

**Keywords:** Capecitabine, Metastatic breast cancer, Progression-free survival, Overall survival

## Abstract

This study aimed to evaluate the efficacy of capecitabine monotherapy for patients with human epidermal growth factor receptor-2 (HER2) normal metastatic breast cancer (MBC). The primary endpoint was progression-free survival (PFS), and secondary endpoints included overall survival (OS) and PFS according to treatment line and estrogen receptor (ER) status. Patients who received capecitabine as monotherapy for HER2 normal MBC from 2010 to 2020 were included in this retrospective study. ER status, treatment line, number of treatments, and dates of progression and death were registered. PFS was defined from capecitabine initiation to progression or any cause of death, and OS until any cause of death. Among 162 patients receiving capecitabine, approx. 70% had ER-positive disease. They received a median of six cycles of capecitabine (range 2–45). The median PFS was 4.3 months, with no significant difference between treatment lines. When analyzing PFS according to ER status, a statistically significant difference was observed between those with ER-positive and ER-negative disease, with a median PFS of 5,3 months versus 2,5 months, respectively (*p* = 0.006). A similar trend was seen for overall survival, with a median OS of 14 months for all patients and 17.8 months versus 7.6 months for patients with ER-positive and ER-negative disease, respectively (*p* ≤ 0.0001). Patients with HER2 normal MBC receiving monotherapy capecitabine had a median PFS of 4.3 months, and a median OS of 14 months. PFS was consistent regardless of treatment line but differed significantly according to ER status.

## Background

Metastatic breast cancer (MBC) remains an incurable disease, with a median overall survival (OS) of three years and a 5-year survival rate of 25% [1]. Treatment aims to optimize disease control by reducing tumor burden while maintaining the highest possible quality of life. Disease prognosis depends on the breast cancer subtype and subsequent available treatment options. When choosing the optimal anticancer therapy agent and dose for each patient, factors such as tumor receptor status, former treatment, comorbidities, personal preferences, and performance status should be considered [1].

Capecitabine is an orally administered fluoropyrimidine derivative with a favorable toxicity profile [2] used as palliative treatment of several solid cancers. It is designed as a prodrug that is converted into its active form 5-fluorouracil (5-FU) in the liver. In the final step in the activation pathway, 5-FU is catalyzed by thymidine phosphorylase (TP), which is present in high concentrations in cancer cells [3]. A study suggests that high thymidylate synthase, among others, is correlated with a shorter progression-free survival (PFS) for capecitabine monotherapy in patients with anthracycline- and taxane-pretreated MBC and it also finds that high TS is more common in patients with triple-negative subtype [4]. A retrospective Japanese study (Amari et al.) conducted in 2009 examined the PFS of capecitabine monotherapy across different lines of treatment of MBC and observed no significant variance among the lines [5].

Most patients with estrogen receptor-positive (ER)/human epidermal growth factor receptor-2 (HER2) normal MBC receive endocrine therapy (ET) combined with cyclin-dependent kinases 4 and 6 inhibitors (CDK4/6i) as first-line treatment [1].

Capecitabine has been used for over two decades in MBC, treating patients with anthracycline- and taxane treatment failure or as a first-line treatment in cases of visceral crisis [6]. However, after the introduction of CDK4/6i, capecitabine is often used in later lines following progression on CDK4/6i and studies regarding the efficacy of capecitabine monotherapy in later treatment lines remains limited.

Therefore, the aim of this study was to investigate the efficacy of capecitabine administered as monotherapy in any treatment line for HER2 normal MBC. The primary endpoint was median PFS, and the secondary endpoints were overall survival and PFS according to treatment line and estrogen receptor status.

## Patients and methods

This retrospective study was conducted at a single institution (Odense University Hospital, Denmark). Patients were eligible if they were diagnosed with HER2 normal MBC and received ≥ 2 cycles of palliative capecitabine as monotherapy between January 1, 2010 and December 31, 2020. For those still alive, the last follow-up day was May 12, 2023.

As part of daily clinical practice, patients received capecitabine for two weeks on treatment and one week off treatment. Response monitoring was performed using either computed tomography (CT), positron emission tomography (PET-CT), magnetic resonance (MR), bone scintigraphy, or a clinical evaluation without strict acquisition to Response Evaluation Criteria in Solid Tumors, RECIST 1.1 [7].

Medical records were screened for data regarding clinical characteristics, pathological information, information about imaging and scans, and medical treatment of MBC before initiating capecitabine and for details on capecitabine treatment. Estrogen receptor and HER2 status were obtained from a metastatic lesion at diagnosis of MBC. Estrogen receptor-positive disease was defined according to ASCO/CAP guidelines with tumor considered ER positive if ≥ 1% expression of ER [8].

Organ involvement was categorized as follows: Patients with central nervous system (CNS) involvement were categorized separately under “CNS”, regardless of any additional spread. Patients with visceral involvement were classified as “visceral” regardless of additional metastases to other sites, except for the CNS. The category “other” encompasses patients with mixed metastasis to soft tissue, lymph nodes, and bone at various sites.

### Statistics

Continuous data were calculated using median and range. Categorical data were reported as frequencies and percentages. PFS, OS, and age at MBC were calculated in Excel, version 16.73. PFS was calculated from the start of capecitabine until the date of progression (PD) or death of any cause. OS was defined as the start of capecitabine until the date of death. XL Stat version 25.1.4 was used when the Kaplan Meier method was applied for estimating PFS according to treatment line and ER status. When calculating PFS, one patient was censored due to death before progression and one patient was censored due to full treatment response. Regarding OS, no patients were censored. A Cox proportional hazard model was made to investigate the difference between ER-positive and ER-negative by hazard ratio.

## Results

### Patient characteristics

**Fig. 1:**
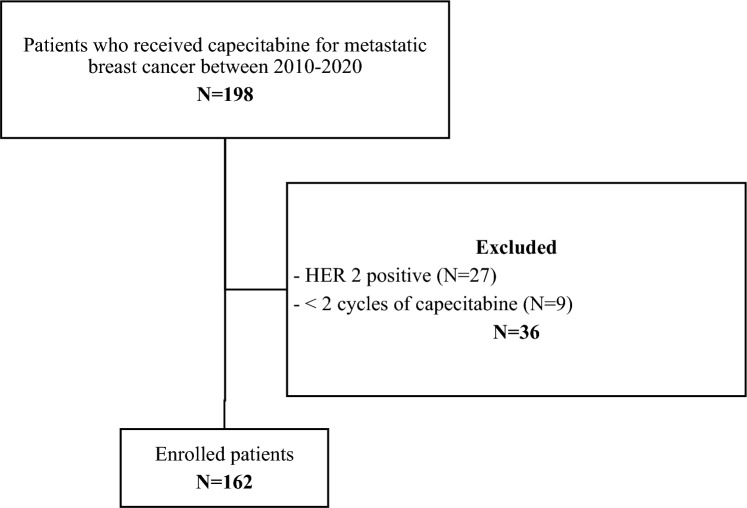
Flowchart of patient inclusion

A total of 162 patients with MBC were included in the study (Fig. 1). The median age was 61.4 years (range 27.0–85.3), and most patients had ER-positive disease (115/162, 71.0%). ER status was negative in 41 patients (25.3%) and unknown in six (3.7%). Most patients (118/162, 72.8%) had visceral metastatic disease at the time of capecitabine initiation, while only 11% (18/162) had bone-only disease.

Approximately 60% of the patients received endocrine therapy before capecitabine and 15.4% received CDK4/6i before capecitabine. In median, patients received one prior treatment line of chemotherapy before initiating capecitabine. Further information about clinical characteristics is shown in Table 1.

**Table 1:** Baseline characteristics at the time of capecitabine treatment

Patients, *N* (%)	162 (100)
Line of capecitabine, median (range)	3.0 (1–9)
Age (median) (range)	61.4 (27.0–85.3)
ER status, *N* (%)	
Positive (1–100%)	115 (71.0)
Negative (0%)	41 (25.3)
Unknown	6 (3.7)
Metastatic site, *N* (%)	
Bone only	18 (11.1)
CNS	10 (6.2)
Visceral	118 (72.8)
Other	16 (9.9)
Performance status, *N* (%)	
0	40 (24.7)
1	41 (25.3)
≥ 2	19 (11.7)
N/A	62 (38.3)
Prior lines of ET, *N* (%)	
0	64 (39.5)
1	36 (22.2)
2	42 (25.9)
≥ 3	20 (12.3)
Prior CDK4/6i, *N* (%)	25 (15.4)
Prior lines of chemotherapy, median (range)	1.0 (0–4)
Prior lines of chemotherapy, *N* (%)	
0	56 (34.6)
1	49 (30.2)
2	41 (25.3)
≥ 3	16 (9.9)
Previous chemotherapy agents^a^, *N* (%)	
Taxane	74 (45.7)
Epirubicin and cyclophosphamide	38 (23.5)
Other^b^	69 (42.6)

*ER* estrogen receptor,* ET* endocrine therapy,* CDK4/6i* cyclin-dependent kinases 4 and 6 inhibitors,* CMF* cyclophosphamidemethotrexate-5fluorouracil

^a^Previous chemotherapy agents did not include neoadjuvant or adjuvant chemotherapy

^b^Vinorelbine, Eribuline, CMF, Carboplatin/gemcitabine and anthracycline monotherapy

### Effect of capecitabine

Patients received a median of six cycles of capecitabine (range 2–45) and received a median of three lines (range 1–9). The most common reason for discontinuing capecitabine was progression (73%, 118/162), while 22% (36/162) stopped treatment due to toxicity, and 5% (8/162) due to other reasons.

**Fig. 2:**
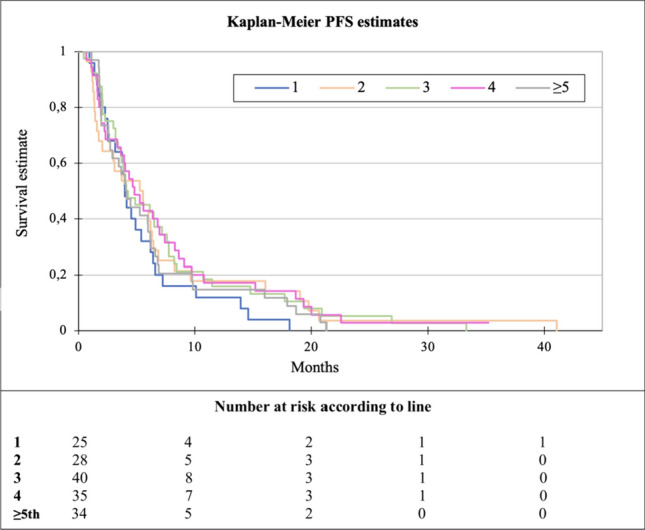
Kaplan–Meier plot of progression-free survival (PFS) according to line of capecitabine

**Fig. 3:**
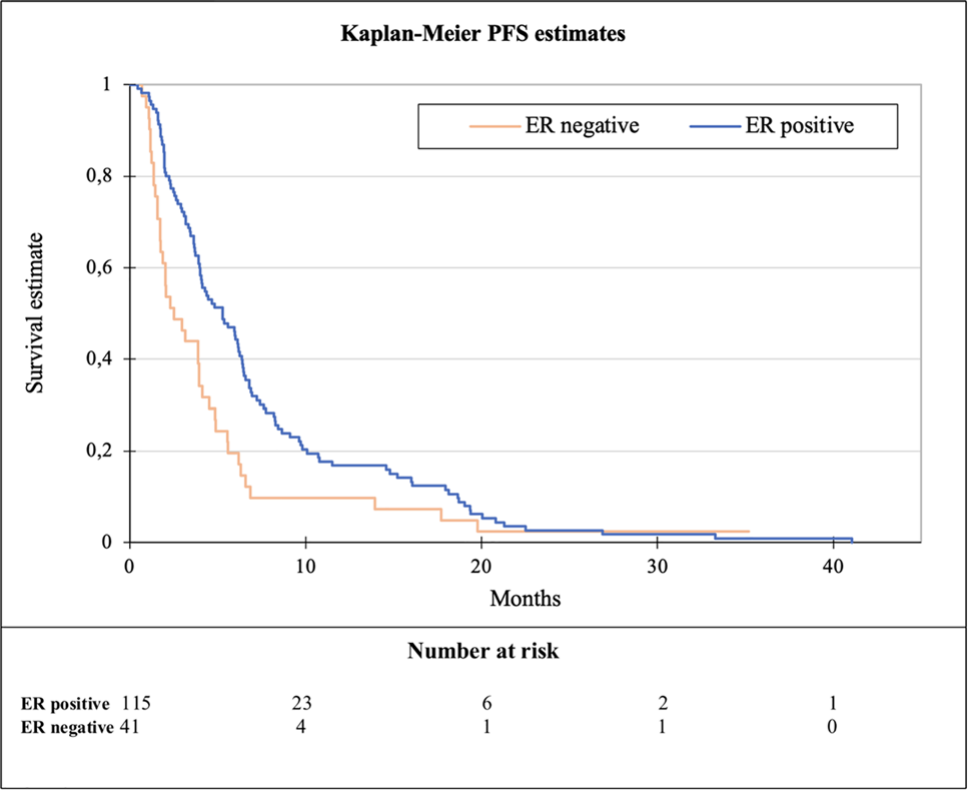
Kaplan–Meier plot of progression-free survival (PFS) according to ER status

**Fig. 4:**
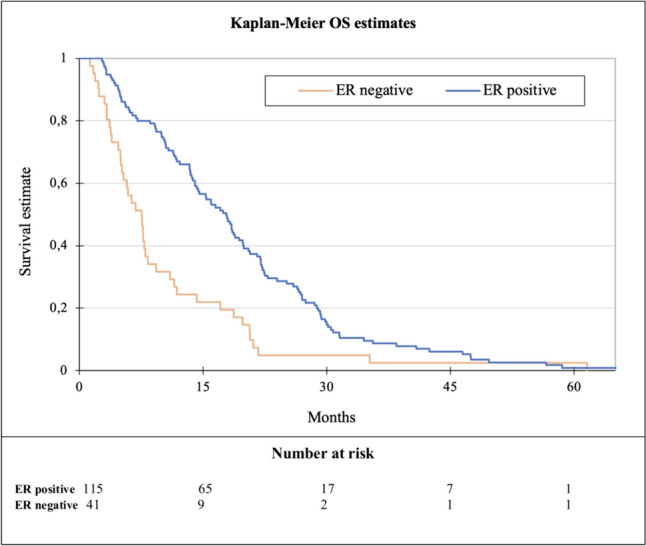
Kaplan–Meier plot of overall survival (OS) according to ER status

The median PFS of capecitabine in any treatment line was 4.3 months (range 0.5–41). No significant difference in the effect of capecitabine was observed when analyzing the different treatment lines (*p* = 0.66, Fig. 2). When dichotomizing patients into those with ER-positive disease (*N* = 115) and those with ER-negative disease (*N* = 41), a statistically significant difference in PFS was observed, being 5.3 months versus 2.5 months, respectively (HR 0.6, *p* = 0.006, Fig. 3). A similar trend was seen for OS, being 17.8 months for ER-positive and 7.6 months for ER-negative disease (*p* ≤ 0.0001, Fig. 4).

A Cox regression analysis revealed a hazard ratio of 0.6 (95% CI, 0–0.9) for ER-positive compared with ER-negative regarding PFS. Furthermore, it was found that the proportion of patients with ER-positive status increased gradually from the first to ≥ the fifth line of capecitabine. More details on capecitabine according to treatment line are shown in Table 2.

**Table 2:** Clinical characteristics and effect of capecitabine according to line of treatment

	Total	1st line	2nd line	3rd line	4th line	≥ 5th line
Number of patients, (%)	162 (100)	25 (15.4)	28 (17.3)	40 (24.7)	35 (21.6)	34 (21.0)
ER status, *N* (%)						
Positive (1–100%)	115 (71.0)	9 (36.0)	13 (46.4)	26 (65.0)	34 (97.1)	33 (97.1)
Negative (0%)	41 (25.3)	16 (64.0)	14 (50.0)	10 (25.0)	1 (2.9)	0 (0.0)
Unknown	6 (3.7)	0 (0.0)	1 (3.6)	4 (10.0)	0 (0.0)	1 (2.9)
Metastatic site, *N* (%)						
Bone only	18 (11.1)	3 (12.0)	1 (3.6)	6 (15.0)	7 (20.0)	1 (2.9)
CNS	10 (6.2)	2 (8.0)	2 (7.1)	2 (5.0)	3 (8.6)	1 (2.9)
Visceral	118 (72.8)	16 (64.0)	19 (67.9)	29 (72.5)	23 (65.7)	31 (91.2)
Other	16 (9.9)	4 (16.0)	6 (21.4)	3 (87.5)	2 (5.7)	1 (2.9)
Number of cycles (median) (range)	6.0 (2–45)	6.0 (2–26)	6.0 (2–45)	6.0 (2–43)	6.0 (2–32)	6.5 (2–29)
PFS (months), median (range)	4.3 (0.5–41)	4.0 (1–18)	5.4 (1–41)	4.1 (1–33)	4.8 (1–35)	4.1 (1–21)
OS (months), median (range)	14.2 (1.3–79.9)	9.3 (1.9–79.9)	11.6 (1.7–58.5)	17.8 (1.3–56.6)	15.3 (2.7–49.7)	16.0 (3.3–46.5)

*ER* estrogen receptor,* PFS* progression-free survival,* OS* overall survival

## Discussion

This retrospective study found a median PFS of 4.3 months for capecitabine given as monotherapy for HER2 normal MBC. The effect of capecitabine was consistent regardless of the treatment line with longer PFS in ER-positive disease compared to ER-negative disease (HR 0.6, 95% CI, 0.0–0.9, *p* = 0.006).

The median PFS of 4.3 months is comparable to other studies. A systematic review from 2011 found a PFS of 18 weeks equivalent to 4.2 months [9]. Furthermore, an open-label phase-two trial found a PFS of 4.6 months [10].

We found no significant difference in median PFS when patients were stratified according to the line of treatment. More patients had ER-negative disease in the early lines compared with later lines, addressing the more aggressive nature of the disease. Another potential explanation could be differences in the distribution of the disease burden between the different treatment lines. Consequently, we hypothesize that patients who received capecitabine in early-line settings were more ill than those who received capecitabine in later lines.

Amari et al. investigated capecitabine and time-to-progression (TTP) distributed by lines. Compared with our study, they found a longer median TTP of 8.5 months for patients in the first and fourth-line treatment settings and 6.5 months for patients in the second and third-line treatment settings [5]. Possible explanations could be that Amari et al. did not account for prior lines of endocrine therapy, and patients in their study did not receive CDK4/6i before capecitabine. In contrast, approx. 15% of patients in our study received CDK4/6i before capecitabine initiation. Further, TTP ignores deaths from other causes than breast cancer, which PFS does not.

We found that the distribution of patients with ER-positive disease increased gradually from the first to the fifth line of capecitabine. This might be due to the better treatment options for ER-positive disease, such as endocrine therapy and CDK4/6i before capecitabine. The skewed distribution between the groups regarding ER status may cause the results since PFS is affected by ER status according to different studies [11, 12]. Due to limited sample sizes within each group, this study did not perform PFS calculations for every treatment line stratified by ER-positive and ER-negative.

Hong et al. suggest that ER-positive can be a useful predictive marker for better PFS to second or later line of capecitabine [2]. However, given the retrospective design of their study, as well as our study, we cannot distinguish between prognostic factors and factors predictive for the effect of capecitabine. Thijssen et al. found that patients with ER-positive disease had a significantly longer TTP compared to patients with ER-negative disease [12]. A recent study by Siddiqui et al. enlightened that thymidylate synthase levels were found to be significantly higher in triple-negative breast cancer [13], which can be the rationale for why ER-positive is linked to a longer TTP. However, this needs confirmation in future studies.

The number of patients who did not receive ET before capecitabine in our study was 64/162 (39.5%). Given the number of 41 patients with ER-negative disease, it is considered a relatively high percentage. Additionally, 25 patients received capecitabine as first-line treatment. It should be noted that the 25 patients who received capecitabine in the first line may have developed MBC during their adjuvant ET, which is not accounted for in this study since only treatment from the time of MBC diagnosis was documented. This may overestimate the number of patients who did not receive ET before capecitabine, which is 64/162 (40%) in this study.

Regarding OS, a median of 14.2 months was found, which resembles the OS in Thijssen et al. of 58 weeks (13.3 months) [12]. When stratified into different lines, it seems as if the best line to receive capecitabine is the third line (17.8 months). This is most likely due to an accumulation of patients with ER-positive disease, who generally have a better prognosis, receiving endocrine therapy and/or CDK4/6i in the first or second treatment line and therefore receive capecitabine in third or later line.

A similar tendency is seen when comparing OS for patients with ER-positive and ER-negative disease, where a significant difference was found.

It is a limitation that our findings are based on a single-center retrospective study with a relatively small sample size. We did not account for adjuvant therapy because the report is made from the time of MBC diagnosis; hence, the patients who relapsed during adjuvant endocrine therapy were not considered. Due to the retrospective study design, it cannot be assured that this study is free of detection bias.

A strength of our study is the strict inclusion of patients with HER2 normal MBC receiving capecitabine as monotherapy in any line, regardless of performance status or comorbidities. Further, we managed to reproduce and validate the findings of earlier studies with a larger sample size [2, 9, 12]. To our knowledge, our study is the first study investigating PFS of late-line capecitabine after the introduction of CDK4/6i.

## Conclusion

The median progression-free survival for patients with HER2 normal MBC receiving capecitabine in any line was 4.3 months, with a median overall survival of 14 months. Progression-free survival was consistent regardless of the line of treatment but differed significantly according to estrogen receptor status, with worse outcomes for patients with estrogen receptor-negative disease.

## Data Availability

Data are available upon reasonable request to the authors.
